# Does the continuation of low-dose acetylsalicylic acid during the perioperative period of thyroidectomy increase the risk of cervical haematoma? A 1-year experience of two Italian centers

**DOI:** 10.3389/fsurg.2022.1046561

**Published:** 2022-11-04

**Authors:** Gian Luigi Canu, Fabio Medas, Federico Cappellacci, Alessio Biagio Filippo Giordano, Francesco Casti, Lucrezia Grifoni, Francesco Feroci, Pietro Giorgio Calò

**Affiliations:** ^1^ Department of Surgical Sciences, University of Cagliari, Monserrato, CA, Italy; ^2^ Department of General and Oncologic Surgery, Santo Stefano Hospital, Prato, PO, Italy

**Keywords:** thyroidectomy, thyroid surgery, antiplatelet drugs, acetylsalicylic acid, cervical haematoma, complications

## Abstract

**Background:**

A growing number of patients taking antiplatelet drugs, mainly low-dose acetylsalicylic acid (ASA) (75–150 mg/day), for primary or secondary prevention of thrombotic events, are encountered in every field of surgery. While the bleeding risk due to the continuation of these medications during the perioperative period has been adequately investigated in several surgical specialties, in thyroid surgery it still needs to be clarified. The main aim of this study was to assess the occurrence of cervical haematoma in patients receiving low-dose acetylsalicylic acid, specifically ASA 100 mg/day, during the perioperative period of thyroidectomy.

**Methods:**

Patients undergoing thyroidectomy in two high-volume thyroid surgery centers in Italy, between January 2021 and December 2021, were retrospectively analysed. Enrolled patients were divided into two groups: those not taking ASA were included in Group A, while those receiving this drug in Group B. Univariate analysis was performed to compare these two groups. Moreover, multivariate analysis was employed to evaluate the use of low-dose ASA as independent risk factor for cervical haematoma.

**Results:**

A total of 412 patients underwent thyroidectomy during the study period. Among them, 29 (7.04%) were taking ASA. Based on the inclusion criteria, 351 patients were enrolled: 322 were included in Group A and 29 in Group B. In Group A, there were 4 (1.24%) cervical haematomas not requiring surgical revision of haemostasis and 4 (1.24%) cervical haematomas requiring surgical revision of haemostasis. In Group B, there was 1 (3.45%) cervical haematoma requiring surgical revision of haemostasis. At univariate analysis, no statistically significant difference was found between the two groups in terms of occurrence of cervical haematoma, nor of the other early complications of thyroidectomy. At multivariate analysis, the use of low-dose ASA did not prove to be an independent risk factor for cervical haematoma.

**Conclusions:**

Based on our findings, we believe that in patients receiving this drug, either for primary or secondary prevention of thrombotic events, its discontinuation during the perioperative period of thyroidectomy is not necessary.

## Introduction

Recent developments in science together with increased access to healthcare have improved the life span of the elderly population suffering from disorders, including cardiovascular and/or cerebrovascular diseases. In this context, a growing number of patients taking antiplatelet drugs, for primary or secondary prevention of thrombotic events, are encountered in daily clinical practice. Consequently, patients receiving these medications are increasingly encountered in every field of surgery (1–6).

Antiplatelet drugs comprise a variety of molecules that act on different platelet receptors and pathways to inhibit their function. The main antiplatelet medication is low-dose acetylsalicylic acid (ASA) (75–150 mg/day). Its antithrombotic effect is due to the irreversible inactivation of cyclooxygenase-1, resulting in a decrease in the synthesis of prostaglandin H2 and thromboxane A2, with consequent inhibition of platelet aggregation (1, 3).

Primary prevention with low-dose ASA is recommended in patients between 40 and 70 years who have an increased risk of thrombotic events but not an increased bleeding risk (7), while indications for secondary prevention are: acute coronary syndromes, stable coronary artery diseases, coronary artery bypass grafting or percutaneous coronary interventions, transcutaneous aortic valve replacement, peripheral arterial disease, carotid disease and ischemic stroke (8–10).

The perioperative management of antiplatelet agents depends on the risk of thrombotic events and bleeding during and after the surgical procedure. Surgeons involved in the care of these patients must decide whether to discontinue or continue these drugs during the perioperative period. In making this decision, it is necessary to consider and weigh the initial indication for their use, from which the possible consequences of their discontinuation depend, the inherent bleeding risk of the surgical procedure to be performed and the impact of bleeding on the patient's overall outcome. Regarding the thrombotic risk, it is also important to remember that surgery itself causes a systemic inflammatory response resulting in the activation of the coagulation system, which predisposes patients to thrombotic events (1–6).

Despite multidisciplinary collaborative efforts, documented in international and national guidelines, recommendations and algorithms, the optimal perioperative management of antiplatelet medications is still a matter of debate (1–6). Moreover, while the bleeding risk due to the continuation of these drugs during the perioperative period has been adequately investigated in several surgical specialties (such as lung, abdominal, spine, orthopedic or urologic surgery), in thyroid surgery it still needs to be clarified (11–18).

The main aim of this study was to assess the occurrence of cervical haematoma in patients taking low-dose acetylsalicylic acid, specifically ASA 100 mg/day, during the perioperative period of thyroidectomy.

## Materials and methods

### Study design and population

This is a multicenter, retrospective, observational study on patients undergoing thyroidectomy between January 2021 and December 2021.

Data were collected from two high-volume thyroid surgery centers in Italy:

- Multi-specialty General Surgery Unit, Cagliari University Hospital, Monserrato (CA);
- General Surgery Unit, Prato Hospital “Santo Stefano”, Prato (PO).

Patients submitted to total thyroidectomy, hemithyroidectomy and completion thyroidectomy were included in this analysis.

Exclusion criteria were: age <18 years, use of antiplatelet drugs different from ASA 100 mg or anticoagulants, simultaneous parathyroidectomy or neck dissection, execution of parathyroid autotransplantation and incomplete data.

Enrolled patients were divided into two groups: those not taking ASA 100 mg/day were included in Group A, while those receiving this medication in Group B.

All patients taking ASA, either for primary or secondary prevention of thrombotic events, continued this drug during the perioperative period.

Demographic and preoperative data, information about the surgical procedure, postoperative stay, histopathological findings and complications were assessed.

### Endpoints

The primary endpoint was to assess the occurrence of cervical haematoma in patients receiving ASA. In the same patients, as secondary endpoints, the other early complications of thyroidectomy (recurrent laryngeal nerve injury, hypoparathyroidism and wound infection), use of drain, operative time and postoperative stay were evaluated.

### Surgical procedure

All patients underwent conventional open thyroidectomy and were euthyroid at the time of surgery.

All operations were performed by surgeons with high experience in thyroid surgery.

Parathyroid glands and recurrent laryngeal nerves were systematically searched and identified.

Energy-based devices (Harmonic Focus—Ethicon, Johnson and Johnson; LigaSure Small Jaw—Medtronic, Covidien Products; and Thunderbeat Open Fine Jaw—Olympus), intraoperative nerve monitoring (IONM) and drain were used according to the preference of the operating surgeon.

Retrosternal goiter was defined as a thyroid in which any part of the gland extended below the thoracic inlet with the patient in the surgical position.

The duration of surgery was estimated, in minutes, from skin incision to skin closure.

### Assessment of complications

Cervical haematomas were distinguished according to whether or not surgical revision of haemostasis was necessary.

Serum calcium and iPTH levels were assayed preoperatively. Postoperatively, iPTH values were assessed 4 h after surgery and on the first and second postoperative days, while serum calcium levels on the first and second postoperative days. Postoperative hypoparathyroidism was defined as iPTH < 10 pg/ml following the operation (normal range = 10–65 pg/ml). Treatment with calcium carbonate (1–3 g/day) and calcitriol (0.5–1.5 µg/day) was administered in patients with diagnosis of postoperative hypoparathyroidism, even in the absence of symptoms.

Preoperative fibrolaryngoscopy was always performed to assess vocal fold mobility. Recurrent laryngeal nerve injury was diagnosed through postoperative fibrolaryngoscopy. After surgery, fibrolaryngoscopy was performed in all cases of suspected recurrent laryngeal nerve injury for loss of signal at IONM or hoarseness. This complication was evaluated considering the number of recurrent laryngeal nerves at risk.

### Statistical analysis

Statistical analyses were performed with MedCalc® 20.110.

Univariate analysis was performed to compare the two groups. Fisher exact test or *χ*^2^ test were utilized for categorical variables. The presence of a normal distribution of continuous variables was assessed using the Shapiro-Wilks test. Based on the results of the latter test, Mann–Whitney *U* test was employed for continuous variables, which were expressed as median and interquartile range (IQR).

Employing multivariate analysis, the use of low-dose ASA was compared with other potential risk factors for cervical haematoma. In this analysis, cervical haematomas requiring surgical revision of haemostasis and those managed conservatively were considered together.

*P*-values were considered statistically significant if <0.05.

## Results

A total of 412 patients underwent thyroidectomy during the study period. Among them, 29 (7.04%) were taking ASA: 15 (51.72%) for primary prevention and 14 (48.28%) for secondary prevention.

Based on the inclusion criteria, 351 patients were enrolled: 322 were included in Group A and 29 in Group B.

Overall, the cervical haematoma rate was 2.56%, all occurring during hospitalization.

### Demographic data, preoperative features and histopathological findings

No statistically significant difference was found in terms of sex, BMI and histopathological findings between the two groups.

Differently, the median age (50.5, IQR 41–61, years in Group A vs. 65, IQR 53–74.25, years in Group B, *P* < 0.001) and rates of high blood pressure (21.12% in Group A vs. 72.41% in Group B, *P* < 0.001) and retrosternal goiter (9.63% in Group A vs. 24.14% in Group B, *P* = 0.026) were significantly greater in Group B than in Group A.

Detailed results are shown in Table 1.

**Table 1:** Demographic data, preoperative features and histopathological findings.

	Total (*n* = 351)	Group A (*n* = 322)	Group B (*n* = 29)	*P-*value
Sex				
Male	91 (25.93%)	81 (25.16%)	10 (34.48%)	0.272
Female	260 (74.07%)	241 (74.84%)	19 (65.52%)	
Age (years, median and IQR)	52 (42–62)	50.5 (41–61)	65 (53–74.25)	<0.001
BMI (kg/m^2^, median and IQR)	25 (22.25–28.69)	24.97 (22.23–28.6)	25.71 (21.89–29.97)	0.563
High blood pressure	89 (25.36%)	68 (21.12%)	21 (72.41%)	<0.001
Retrosternal goiter	38 (10.83%)	31 (9.63%)	7 (24.14%)	0.026
Histological diagnosis				
Graves’ disease	33 (9.40%)	31 (9.63%)	2 (6.90%)	1.000
Other benign diseases	139 (39.60%)	129 (40.06%)	10 (34.48%)	0.556
Malignancy	188 (53.56%)	170 (52.80%)	18 (62.07%)	0.338

IQR, interquartile range; BMI, body mass index.

### Information about the surgical procedure and postoperative stay

No statistically significant difference was found as regards type of surgery (total thyroidectomy, hemithyroidectomy and completion thyroidectomy), use of intraoperative nerve monitoring, use of energy-based devices, use of drain, operative time and postoperative stay between the two groups.

Detailed results are reported in Table 2.

**Table 2:** Information about the surgical procedure and postoperative stay.

	Total (*n* = 351)	Group A (*n* = 322)	Group B (*n* = 29)	*P-*value
Type of surgery				
TT	220 (62.68%)	203 (63.04%)	17 (58.63%)	0.470
HT	112 (31.91%)	103 (31.99%)	9 (31.03%)	
CT	19 (5.41%)	16 (4.97%)	3 (10.34%)	
Use of IONM	338 (96.30%)	312 (96.89%)	26 (89.66%)	0.083
Use of EBD	351 (100%)	322 (100%)	29 (100%)	–
Use of drain	273 (77.78%)	249 (77.33%)	24 (82.76%)	0.501
Operative time (minutes, median and IQR)	75 (60–85)	75 (60–85)	75 (50–101.25)	0.814
Postoperative stay (days, median and IQR)	2 (1–3)	2 (1–3)	2 (1–4)	0.235

TT, total thyroidectomy; HT, hemithyroidectomy; CT, completion thyroidectomy; IONM, intraoperative nerve monitoring; EBD, energy-based device; IQR, interquartile range.

### Complications

In Group A, there were 4 (1.24%) cervical haematomas not requiring surgical revision of haemostasis, 4 (1.24%) cervical haematomas requiring surgical revision of haemostasis, 50 (15.53%) cases of postoperative hypoparathyroidism, 14 (2.67%) unilateral RLN lesions and 4 (1.24%) wound infections. No bilateral recurrent laryngeal nerve injury occurred in this group.

In Group B, there were 1 (3.45%) cervical haematoma requiring surgical revision of haemostasis, 3 (10.34%) cases of postoperative hypoparathyroidism and 2 (4.35%) unilateral RLN lesions. No cervical haematoma not requiring surgical revision of haemostasis, bilateral recurrent laryngeal nerve injury or wound infection occurred in this group.

No statistically significant difference was found in terms of complications between the two groups.

Detailed results are shown in Table 3.

**Table 3:** Complications.

	Total (*n* = 351)	Group A (*n* = 322)	Group B (*n* = 29)	*P-*value
Cervical haematoma				
No surgical revision	4 (1.14%)	4 (1.24%)	0	1.000
Surgical revision	5 (1.42%)	4 (1.24%)	1 (3.45%)	0.352
Postoperative hypoparathyroidism	53 (15.10%)	50 (15.53%)	3 (10.34%)	0.594
RLN injury^a^				
Unilateral	16 (2.80%)	14 (2.67%)	2 (4.35%)	0.374
Bilateral	0	0	0	–
Wound infection	4 (1.14%)	4 (1.24%)	0	1.000

RLN, recurrent laryngeal nerve.

^a^ Considering the number of recurrent laryngeal nerves at risk.

### Multivariate analysis

In this analysis, only BMI was found to be an independent risk factor for cervical haematoma. Specifically, higher BMI values were found to be statistically significantly correlated with the development of this complication (RC 0.164, OR 1.178, 95% CI, 1.037–1.338, *P* = 0.012).

Differently, the use of low-dose ASA and the other variables analyzed did not prove to be independent risk factors.

Detailed results are reported in Table 4.

**Table 4:** Multivariate analysis of potential risk factors for cervical haematoma.

	Regression coefficient	Odds ratio	95% CI	*P–*value
Male sex	0.007	1.007	0.167–6.070	0.994
Age	−0.020	0.980	0.919–1.046	0.546
BMI	0.164	1.178	1.037–1.338	0.012
High blood pressure	1.288	3.626	0.631–20.849	0.149
Retrosternal goiter	−20.217	<0.001		0.998
Use of low-dose ASA	0.649	1.913	0.194–18.836	0.578
Type of surgery				
TT	1.000	1.000	Reference	
HT	−0.191	0.826	0.125–5.457	0.843
CT	−19.612	<0.001		0.999
Use of IONM	−19.844	<0.001		0.999
Use of drain	0.071	1.073	0.142–8.104	0.945
Operative time	−0.007	0.993	0.961–1.026	0.663
Histological diagnosis				
Graves’ disease	−20.408	<0.001		0.999
Other benign diseases	−20.990	<0.001		0.999
Malignancy	−20.762	<0.001		0.999

BMI, body mass index; ASA, acetylsalicylic acid; TT, total thyroidectomy; HT, hemithyroidectomy; CT, completion thyroidectomy; IONM, intraoperative nerve monitoring.

## Discussion

Thyroidectomy is the most frequently performed surgical procedure in endocrine surgery, for the treatment of both benign diseases and malignancy (19–22).

In thyroid surgery, achieving accurate haemostasis is essential to prevent the occurrence of cervical haematoma (23). Moreover, avoiding bleeding in the surgical field during the operation, allowing adequate vision of the anatomical structures, is important to avert the occurrence of the other complications of thyroidectomy (recurrent laryngeal nerve injury and hypoparathyroidism) (24–29).

Cervical haematoma is an uncommon complication after thyroid surgery, occurring in 0.3%–4.2% of cases (30–47). It mostly occurs in the first 6 h after the surgical procedure, while after 24 h it is quite infrequent (31, 33, 34, 36, 41, 42, 44–46). This complication is potentially life-threatening as it can lead to acute airway obstruction, through direct compression or venous congestion resulting in significant airway oedema, which can be followed by hypoxic brain injury or even death after a few minutes, if no prompt intervention is taken (48). Cervical haematoma with associated airway compromise occurs after thyroidectomy in 0.9%–2.1% of cases and it is estimated that one-quarter of these patients require an immediate life-saving clot evacuation and 0.3% a tracheostomy (30, 31, 37). Therefore, this complication is a major concern for surgeons after thyroidectomy. However, it is important to note that previous studies suggested that 46% of cervical haematomas can be managed non-operatively (33).

Since this complication is infrequent, the assessment of factors associated with its occurrence is challenging. However, several risk factors were reported in the literature: male gender, older age, active smoking, high blood pressure, active use of anticoagulant drugs, extent of surgery (bilateral thyroidectomy, neck dissection), previous thyroid surgery, use of drain, use of haemostatic agents, low surgeon experience, large thyroid gland, retrosternal goiter, Graves' disease, thyroid malignancy (31–37, 41–43, 45, 46).

As previously mentioned, the effect of antiplatelet medications on the occurrence of cervical haematoma still needs to be clarified. To date, there are few studies in the literature on this topic. Furthermore, it is important to note that in some of these investigations antiplatelet agents are discontinued before surgery, while in others the exact perioperative management of these drugs is not specified (31, 33, 34, 39–42, 45). Ultimately, there are only two studies that evaluate the impact of the continuation of antiplatelet medications during the perioperative period of thyroid surgery (46, 47).

The main aim of this study was to assess the occurrence of cervical haematoma in patients taking low-dose acetylsalicylic acid, specifically ASA 100 mg/day, during the perioperative period of thyroidectomy. Moreover, in the same patients, as secondary endpoints, the other early complications of thyroidectomy (recurrent laryngeal nerve injury, hypoparathyroidism and wound infection), use of drain, operative time and postoperative stay were evaluated.

Regarding the type of surgical procedure performed, all patients in this study underwent conventional open thyroidectomy. Moreover, in order to obtain a homogeneous comparison, considering that no patient receiving ASA underwent simultaneous parathyroidectomy or neck dissection or parathyroid autotransplantation, these procedures were considered as exclusion criteria.

The comparison between the two groups did not reveal a statistically significant increase in the occurrence of cervical haematoma, nor of the other early complications of thyroidectomy, in patients taking ASA. No statistically significant difference was also observed in terms of use of drain, operative time and postoperative stay. Moreover, the use of low-dose ASA did not prove to be an independent risk factor for cervical haematoma.

Concerning our result on the occurrence of cervical haematoma, it is interesting to note that in patients taking ASA it was not increased even though they had an older age and greater rates of retrosternal goiter and high blood pressure, which are well-documented independent risk factors for this complication (31–37, 41–43, 45, 46).

The other two studies evaluating the effects of the continuation of antiplatelet agents during the perioperative period of thyroid surgery were conducted by Campbell et al. and Raggio et al. (46, 47).

In the study by Campbell et al. (46) the use of antiplatelet or anticoagulant drugs during the perioperative period of thyroidectomy was found to be an independent risk factor for the occurrence of cervical haematoma. However, their result is limited by the fact that, due to the small number of patients taking antiplatelet or anticoagulant medications, the multivariate analysis was performed by grouping together patients receiving nonsteroidal anti-inflammatory drugs, ASA, clopidogrel or warfarin/low-molecular-weight heparin (LMWH). Moreover, considering the univariate analysis, it is important to note that the increased occurrence of cervical haematoma appears to be determined by the use of clopidogrel or warfarin/LMWH rather than ASA.

Raggio et al. (47), based on the results of their analysis, stated that ASA can be continued in the perioperative period of thyroid surgery without increasing intraoperative bleeding. As regards the occurrence of cervical haematoma and RLN injury, these authors consider their findings inconclusive due to the small sample size and number of outcome events.

About these two studies, it is important to point out that in the analysis of Campbell et al. (46) the dosage of ASA is not specified, while in that of Raggio et al. (47) patients taking ASA 81 mg/day and those receiving ASA 325 mg/day were included in the same group, in which there are also some patients using ASA in combination with other antiplatelet and/or anticoagulant drugs (clopidogrel and warfarin).

Our study has some limitations. First of all, it is based on a retrospective analysis, thus at risk of bias. The second limitation consists in the limited number of patients taking ASA. This last condition, together with the low occurrence of cervical haematoma, strongly hinders the achievement of a statistical power suitable for an accurate evaluation of this complication (result of the post-hoc power analysis: 7.5%). The third limitation is that it was not possible to specify whether intraoperative haemostatic agents were used, as this information was not available in patients' medical records. In this regard, however, it is important to emphasize that intraoperative haemostatic agents are rarely utilized in both centres.

## Conclusion

Continuation of ASA 100 mg/day during the perioperative period of thyroidectomy did not increase the occurrence of cervical haematoma and the other early complications of thyroid surgery (recurrent laryngeal nerve injury, hypoparathyroidism and wound infection). Based on our findings, we believe that in patients receiving this drug, either for primary or secondary prevention of thrombotic events, its discontinuation during the perioperative period of thyroidectomy is not necessary.

However, given the limitations of our and the other two analyses, further prospective studies with larger populations are needed to better investigate this topic.

## Data Availability

The raw data supporting the conclusions of this article will be made available by the authors, without undue reservation.
